# Analysis of Variations in Maxillary Second Molar Buccopalatal Inclination in Angle’s Class I, Angle’s Class III Malocclusion, and Unilateral Cleft Lip and Palate (UCLP) Cases: A Comparative Observational Study

**DOI:** 10.7759/cureus.31746

**Published:** 2022-11-21

**Authors:** Jeni Ann Mathew, Ranjit Kamble, Sumukh Nerurkar, Dhwani Suchak, Japneet K Kaiser

**Affiliations:** 1 Department of Orthodontics, Sharad Pawar Dental College, Datta Meghe Institute of Medical Sciences (DMIMS) Deemed University, Wardha, IND

**Keywords:** stable results, postsurgical orthodontics, presurgical orthodontics, planning, unilateral cleft lip and palate, malocclusion, force, second molar, occlusion, inclination

## Abstract

Introduction

Cleft of the lip and/or the palate is commonly inherited defect which involves cleft of lip and palate. Maxillary second molar inclination has been studied in various malocclusion. Every abnormality in body is compensated to some extend by compensation. Our objectives were to evaluate maxillary second molar inclination in Angle's Class I, Class III malocclusion, and unilateral cleft lip and palate (UCLP) patients in transverse plane and to compare variation in maxillary second molar inclination in Angle's Class I, Class III malocclusion, and UCLP cases buccopalatally in transverse plane.

Material and method

Model of 45 subjects were analyzed. The maxillary second molar inclination was measured using aid of protractor fixed on the surveyor. Axis under consideration was the axis along the long axis of the central fossa of maxillary second molar. Various standardization regarding measurement of second molar inclination were set. The maxillary second molar inclination was compared among 3 groups Angle's Class I, Class III malocclusion, and UCLP cases.

Results

Data was analyzed using one-way analysis of variance (ANOVA) and post-hoc Tukey test. There was a significant difference between inclination of maxillary second molar when Angle's Class I malocclusion was compared with Angle's Class III malocclusion and UCLP cases (p = 0.003 and p = 0.011, respectively). There was not a significant difference between Angle's Class III malocclusion and UCLP cases (p = 0.87).

Conclusion

Amongst Class III patients and UCLP patients the inclination of maxillary second molar had greater buccal inclination. Maxillary second molar correction would alleviate the effect of deleterious force on periodontium and bone generated by malpositioned teeth.

## Introduction

Cleft lip (CL) and cleft palate (CP) are abnormalities found in general population. Patients may suffer from cleft lip and palate (CLP) or express individually. Males are effected more by CLP, and in females, CP is more prevalent. Reddy et al. quoted that incidence is 28,600 in India, which implies that three children are diagnosed with cleft each hour [1]. The incidence have chances of being higher because in India, due to various reasons, the detection of abnormality in population is not done by a single agency in the country, and survey regarding collection of data regarding the incidence suffers a setback [1]. Facial region can be affected in different region or different combination. In cleft subjects, the structure that is generally affected is maxilla. Etiology can be genetic in origin, environmental, or both. Prevalence of maxillary constriction is common, and Class III tendency and expression is common in cleft patients. The existing condition is exaggerated by constriction caused by surgical procedure as expression of growth is affected. Esthetic is often ignored by patients when skeletal deformity is mild. The deformity is generally compensated for and if it is accompanied by orthodontic intervention, it can efficiently camouflage the skeletal disparity [2].

The severity of manifestation of Class III malocclusion in cleft patients varies. Occlusal plane is affected in patients, and loading of condyle is affected across the temporomandibular joint. Existing condition is exaggerated by constriction caused by surgical procedure as expression of growth is affected. The team providing intervention consists of surgeons, orthodontists, pedodontists, speech therapists, etc [3]. Malposition of tooth is generally accompanied by supraeruption of teeth. The transmission of force is altered from normal axis, causing uneven loading of force. Intercuspation of teeth is of importance in stability of orthodontic intervention. Second molar is often not involved in treatment due to difficulty in bonding or banding of second molar [4]. 

CLP and accompanying abnormalities worsen as age advances. They hamper the normal function and esthetics. It affects the growth of facial region and alignment of dentition. The first step in treatment involves diagnosis, and errors in diagnosis and planning of treatment hampers the results in terms of retention and stability. Orthodontic intervention is of crucial importance at different phases of treatment. The earliest intervention is presurgical orthopedics and intermediate stages involve achieving transverse occlusion. Preparation of dentition in all planes (transverse, vertical, sagittal) in presurgical and postsurgical orthodontics is mandatory for retention and stability. Vigilant treatment planning at proper time is crucial for maximum achievement of results [5].

Incidence of bilateral cleft lip and palate (BCLP) is less than unilateral cleft lip and palate (UCLP). Thus, our study involved cleft patients having UCLP because they constitute major sufferer of cleft deformity [6]. The study of genetic and external influence on CLP patients in terms of prognosis of treatment should be analyzed. The studies on maxillary second molar are limited. Hence, maxillary second molar inclination in Angle's Class I, Class III malocclusion, and UCLP cases were studied [7].

## Materials and methods

The ethical approval for the study was granted by Datta Meghe Institute Medical Ethical Committee with Ref. No. DMIMS(DUYIEC/2020-21/256). The observational study was performed in the Department of Orthodontics and Dentofacial Orthopedics, Sharad Pawar Dental College, Datta Meghe Institute of Medical Sciences, Wardha, India. Patients who had visited the outpatient department (OPD) of Department of Orthodontics and Dentofacial Orthopedics, Sharad Pawar Dental College, Datta Meghe Institute of Medical Sciences, India were selected. Patients with Angle's Class I, Class III malocclusion, and UCLP patients were randomly selected from patients who had reported. Subjects with complete permanent set of dentition were included. Patient who had no history of orthodontic intervention were included. UCLP patients who had undergone surgery were selected. Maxillary second molar with incomplete eruption or had not erupted or missing were excluded.

**Figure 1:**
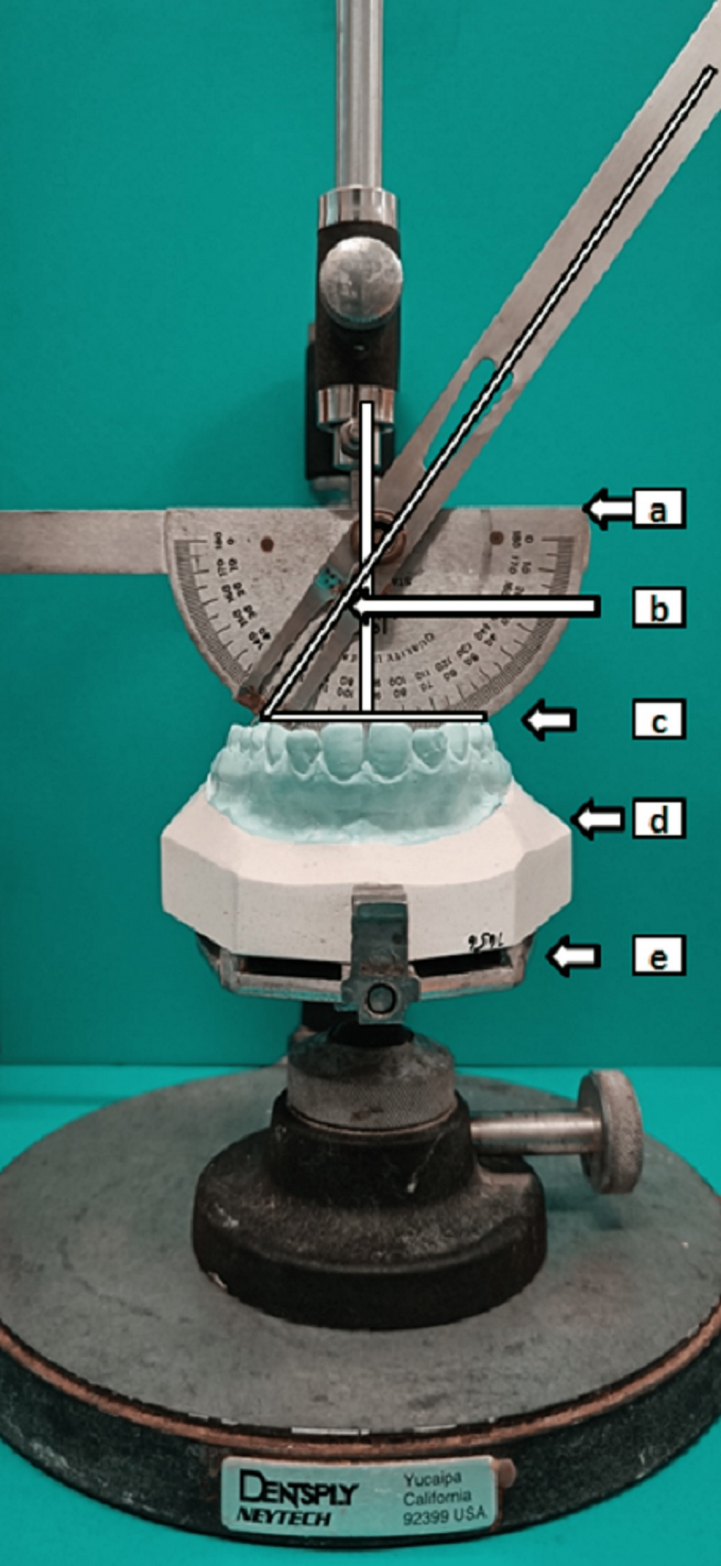
Protractor fixed on surveyor to measure second molar inclination with standardized norms. (a) Plane of protractor; (b) AoI; (c) Occlusal plane; (d) Maxillary cast; and (e) Surveying table. AoI: Angle of inclination

Procedure for measurement of maxillary second molar inclination in Angle's Class I, Class III malocclusion, and UCLP patients was standardized. Impressions of selected subjects were taken. Cast of subjects were poured in dental stone and base poured. Surveying table was utilized, protractor was assembled on it. Norms set was occlusal plane (Figure 1(c)) adjusted parallel to floor. Planes under consideration are occlusal plane and protractor baseline plane (Figure 1(a)). The long axis of the tooth along central fossa was measured. Inclination of crown or angle of inclination (AoI) (Figure 1(b)) is formed between perpendicular line to occlusal plane and the long axis along central fossa. Angle was measured as positive if teeth were inclined buccally and negative if palatally inclined. The measurement was done on both sides and average values were considered.

## Results

**Figure 2:**
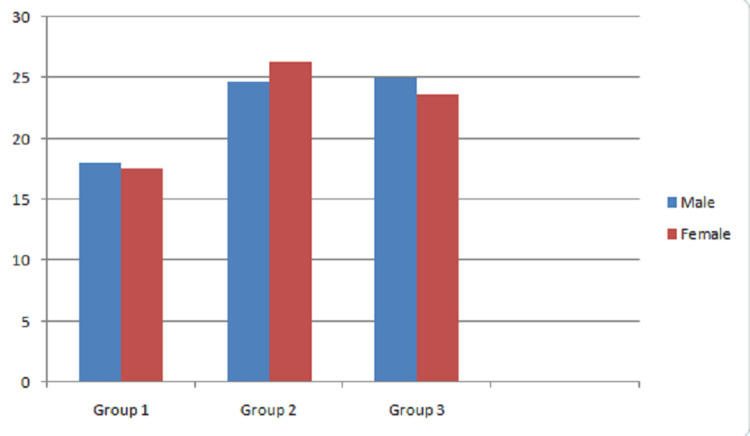
Mean of AoI in male and female participants in Group 1 (Angle's Class I malocclusion), Group 2 (Angle's Class III malocclusion), and Group 3 (UCLP patients). Aoi: Angle of inclination; UCLP: Unilateral cleft lip and palate

In the observational study, 45 pretreatment models were analyzed. The models surveyed included 21 males and 24 females. Average age of male subjects was 17 years and females was 19 years. Each group included had 15 study model of Angle's Class I, Class III malocclusion, and UCLP patients. Inclination measurement was done on both sides and mean value was taken as inclination value of each model. AoI in males and females was measured as depicted in Figure 2. Mean value of AoI was measured (Table 1): mean value of Angle's Class I malocclusion was 17.7 years and Class III malocclusion was 25.6 years. Comparative analysis of Angle's Class I malocclusion and Class III malocclusion patients had significant difference (p = 0.003). Mean value of Angle's Class III malocclusion was 25.6 years and UCLP patients was 24.5 years. Comparative analysis of inclination of Angle's Class III malocclusion and UCLP patients had significant difference (p = 0.011) and there was not a significant difference between Angle's Class III malocclusion and UCLP patients when inclination was compared (p=0.87) (Table 2).

**Table 1:** AoI of second molar in Group 1 (Angle's Class I malocclusion), Group 2 (Angle's Class III malocclusion), and Group 3 (UCLP patients). AoI: Angle of inclination; UCLP: Unilateral cleft lip and palate

	Group 1		Group 2		Group 3	
	Mean	SD	Mean	SD	Mean	SD
AoI	17.73	5.86	25.6	5.47	24.47	6.81

**Table 2:** Comparison of AoI among Group 1 (Angle's Class I malocclusion), Group 2 (Angle's Class III malocclusion), and Group 3 (UCLP patients). AoI: Angle of inclination; UCLP: Unilateral cleft lip and palate

	Group 1 vs Group 2			Group 1 vs Group 3			Group 2 vs Group 3		
	Mean		p value	Mean		p value	Mean		p value
	Group 1	Group 2		Group1	Group 3		Group 2	Group3	
AoI	17.73	25.6	p = 0.003	17.73	24.47	p = 0.011	25.6	24.47	p = 0.866

## Discussion

Deformities in the face vary in form: dental and skeletal components are affected differently and prognosis of treatment varies with the severity of the defect. Orthodontic treatment involves an equal emphasis on treatment planning and execution. The presurgical and postsurgical orthodontics involves considerations of biomechanics with respect to surgical requirements in the patients. Orthodontics and orthognathic surgery in combination help in enhancing function as well as esthetics of patients with CLP. Cases in which presurgical or postsurgical orthodontics are required are executed by an orthodontist, and planning is done by a multidisciplinary team. In the proper prognosis of orthognathic surgery, presurgical orthodontics plays a crucial role. Nature has a way of compensating and masking abnormalities. Resolving the compensation is the goal of presurgical orthodontics. When there is a lapse in treatment provided because of a lack of understanding, it can lead to compromised results [8-10].

According to Walton and Houston et al., occlusion can be deranged when teeth in any space of plane are in malrelation, which means the axis of tooth marks is important in occlusion. The increased second molar buccolingual inclination in Class III and UCLP patients can lead to a change in forces transmitted along the second molar to the bone. However, correction of second molar inclination can lead to increased harmony of force transmission to bone [11].

Levine et al. observed in Class III patients that second molars are inclined buccally or palatally. The findings of our study are also in agreement with Levine et al., in that the second molar inclination was affected in variable measures in Angle's Class I, Class III malocclusion, and UCLP cases. Angulation and inclination of posterior teeth are of paramount importance in patients who had undergone orthognathic surgeries to prevent the subsequent deleterious effect [4]. Arriola-Guillén et al. observed discrepancy of angulation and inclination of teeth in the maxillary posterior segment had a substantial impact on skeletal open bite patients [12]. Khechoyan et al. emphasized the importance of analysis of dental and facial deformity for the success of orthognathic surgery. The variable degree of inclination of the second molar needs to be studied to correct it, according to need to correct the force vector [13].

The rationale behind this study was an analysis of second molar inclination in Angle's Class I, Class III malocclusion, and UCLP patients. As per the findings of past studies by Craddock et al., absence of opposing teeth in the lower arch has a concomitant finding of buccally placed teeth [14]. Our current study also found that buccal inclination of the second molar was common among subjects examined. Angle's Class III patients had larger buccal inclination than Angle's Class I malocclusion patients. Molars were buccally tipped. It is in agreement with our current study where it was concluded that Angle's Class III patients had a increased buccal inclination of the second molar than Angle's Class I malocclusion patients. According to a study by Golshah et al., skeletal Class III patients had more buccal inclination than Class I patients which is comparable to our study [15]. The research conducted by Levine et al. established that buccopalatal inclination of the maxillary second molar had increased inclination in Class III patients than in Class I skeletal pattern patients. The maxillary second molar inclination under consideration has the different force transmission to bone. Study should expand towards effect of force vector on bone [7].

## Conclusions

There was no significant difference in maxillary second molar inclination amongst Angle's Class III malocclusion subjects and UCLP patients. Evaluation of maxillary second molar inclination would create a better understanding of occlusion and better treatment planning. This would promote better stable results post orthognathic surgery in particular and add to retention and relapse of orthodontic treatment in general by aiding in providing better stable occlusion. Every possible effort should be taken by a multidisciplinary team to elevate the functional disability and provide treatment to enhance better function amongst UCLP patients.
